# Successful Outcome of Mother and Baby in a Woman With Severe COVID-19 Pneumonia in the Third Trimester of Pregnancy Who Required Extracorporeal Membrane Oxygenation Therapy

**DOI:** 10.7759/cureus.34627

**Published:** 2023-02-04

**Authors:** Fumio Asano, Shinji Tanigaki, Yasunori Sato, Chie Kobayashi, Yoichi Kobayashi

**Affiliations:** 1 Department of Obstetrics and Gynecology, Kyorin University School of Medicine, Tokyo, JPN

**Keywords:** severe respiratory failure, p/f ratio, pregnancy, extracorporeal membrane oxygenation support, covid-19

## Abstract

Some studies have reported increased severe coronavirus disease (COVID-19) infection in the third trimester of pregnancy. Therefore, prenatal care in the third trimester requires careful judgment. It has been reported that extracorporeal membrane oxygenation (ECMO) therapy is useful for severe coronavirus disease 2019 (COVID-19) pneumonia; however, the optimal timing for the initiation of ECMO is controversial because the risks and benefits to the mother and fetus require careful consideration. We report a good outcome for mother and baby in a pregnant woman with severe COVID-19 pneumonia at 29 weeks gestation, who underwent urgent delivery and required ECMO therapy.

A 34-year-old woman tested positive for COVID-19 at 27 weeks gestation. Despite treatment with remdesivir and prednisolone, her respiratory condition worsened. Consequently, she underwent emergent endotracheal intubation at 28 weeks and 2 days. Although the PaO_2_/FiO_2_ (P/F ratio) improved temporarily after endotracheal intubation, her respiratory condition progressively worsened. At 29 weeks gestation, an emergency cesarean section was performed and ECMO was initiated the next day. Although hematoma was observed after ECMO initiation, her respiratory condition improved. She was discharged home 54 days after the cesarean delivery without any complications. The neonate was intubated and transferred to the neonatal intensive care unit and was ultimately discharged home without any complications.

Considering the risks and benefits of ECMO for the mother and fetus in the third trimester, ECMO should be initiated after delivery for better outcomes. The P/F ratio may be useful for an appropriate decision regarding delivery and the initiation of ECMO.

## Introduction

Some studies have reported increased severe coronavirus disease 2019 (COVID-19) in the third trimester of pregnancy [1]. Nicola et al. reported that 75% of all the pregnancies admitted for COVID-19 in their study were in the third trimester, indicating that the third trimester of pregnancy increased the risk of hospitalization due to COVID-19 [2]. In this report, there were maternal and perinatal deaths due to severe COVID-19 infection. Therefore, prenatal care in the third trimester requires careful judgment.

Subsequently, there have been multiple reports of pregnant women infected with severe COVID-19. While it has been reported that extracorporeal membrane oxygenation (ECMO) therapy is useful for severe COVID-19 pneumonia, the optimal timing for the initiation of ECMO and whether or not to continue the pregnancy in women with severe COVID-19 pneumonia remains unclear, as the risk and benefit to both mother and fetus need to be considered [3-11].

We report a successful outcome for both mother and baby in a pregnant woman with severe COVID-19 pneumonia at 29 weeks gestation, who underwent urgent delivery and required ECMO therapy.

## Case presentation

A 34-year-old, gravida-1, para-0 woman with no significant medical history presented to our institution. She was not vaccinated against severe acute respiratory distress syndrome coronavirus 2 (SARS-CoV-2) and tested positive for COVID-19 at 27 weeks and 0 days after the initial onset of symptoms of dry cough and fever. She was first admitted to a different hospital.

On admission, she required oxygen inhalation therapy with a nasal cannula to achieve an oxygen saturation (SpO2) of 95%. In addition, a prophylactic dose of anticoagulants was started with unfractionated heparin (10,000 U/day). Although her condition was stable with a nasal cannula for a few days, she developed a fever of 39℃ and worsening oxygen saturation and dyspnea; thus, at 27 weeks and 5 days, she was treated with an oxygen mask, remdesivir (first dose, 200 mg/day; further doses, 100 mg/day), and prednisolone (40 mg/day). However, her respiratory condition rapidly worsened the next day, and her SpO2 level was 88% with an oxygen mask at 10 L/min with a reservoir bag; she was transferred to our institution at 28 weeks and 0 days.

**Figure 1:**
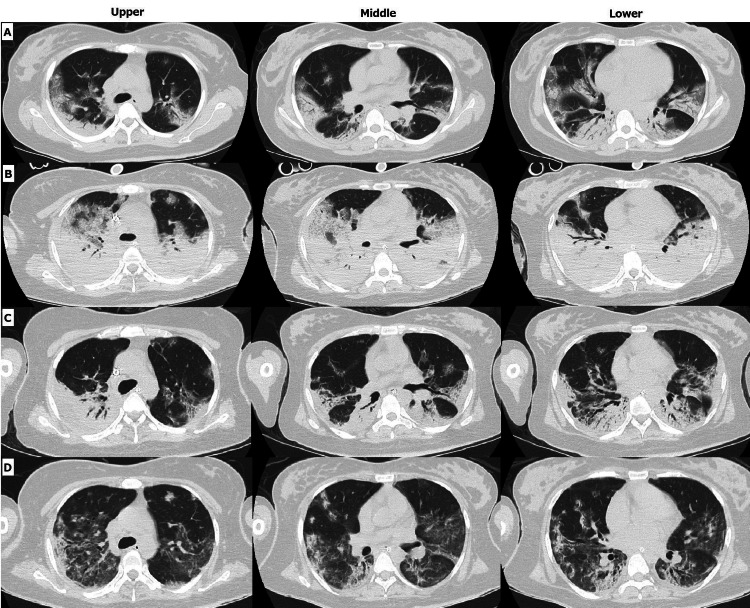
Chest computed tomography (CT) of the patient A. Chest CT on admission (28 weeks, 0 days). Pulmonary consolidation was observed in bilateral dorsal lungs. B. Chest CT on initiation of VV-extracorporeal membrane oxygenation (ECMO) (POD1). Diffuse-distributed ground-glass shadows in the bilateral lung fields were observed. The P/F ratio worsened, requiring VV-ECMO. C. Chest CT on laparotomy (POD9). The ground-grass shadows in the bilateral lungs tended to be reduced. D. Chest CT 1 day before extubation (POD15). The ground-glass shadows in the bilateral lungs were mostly absorbed. CT, computed tomography; VV, venovenous; POD, postoperative day

On arrival, chest computed tomography (CT) revealed an infection in the bilateral dorsal lung (Figure 1A).

With worsening oxygen saturation and dyspnea, SpO2 was 97% with an oxygen mask at 10 L/min with a reservoir bag. This treatment was continued.

The next day, with an increasing need for oxygen, she was placed on a high-flow nasal cannula (HFNC) and prone positioning. However, her condition continued to worsen with HFNC (40 L/min FiO2:1.0); thus, she was treated with tocilizumab (600 mg) and underwent emergent endotracheal intubation at 28 weeks and 2 days.

Moreover, obstetric ultrasonography revealed an estimated fetal weight of 1309 g (+0.95 SD). Additionally, the condition of the fetus was good, the placenta was localized on the posterior wall, the amniotic fluid volume was normal, and no signs of intrauterine infection were observed. Thereafter, the fetal condition was assessed regularly with obstetric ultrasonography and a non-stress test (NST), and fetal well-being remained good until delivery. Considering the long-term neurodevelopmental outcomes after antenatal corticosteroid therapy, she was treated with prednisolone but not dexamethasone. After initiation of endotracheal intubation, in preparation for delivery, prednisolone was changed to dexamethasone (6.6 mg/day) for fetal lung maturation, and MgSO4 was used for neuroprotection.

The PaO2/FiO2 (P/F ratio) was >180 immediately after endotracheal intubation, and the ratio improved to 330 at 28 weeks and 4 days. However, the P/F ratio decreased to 100 at 28 weeks and 5 days.

**Figure 2:**
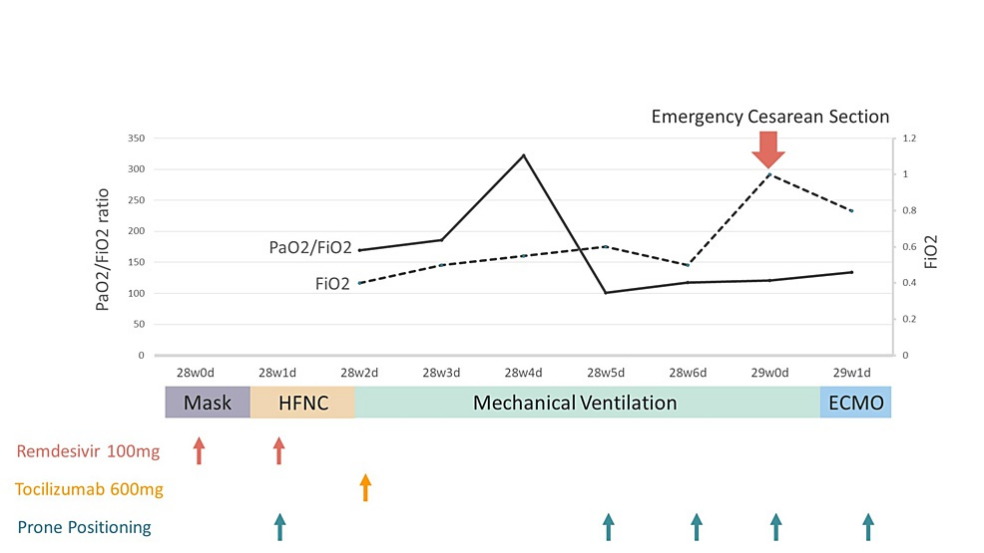
Time course of the patient regarding lung conditions and therapeutic interventions from arrival at our institution to the initiation of VV-ECMO VV-ECMO, venovenous extracorporeal membrane oxygenation

As her respiratory condition progressively worsened, her refractory hypoxic respiratory failure required venovenous (VV)-ECMO. Considering the benefits and risks for the mother and fetus, the decision was made to proceed with an emergency cesarean section before the initiation of VV-ECMO (Figure 2).

At 29 weeks and 0 days, an emergency cesarean section was performed uneventfully, resulting in the delivery of a baby boy (1479 g; UmApH, 7.290; Apgar score, 3 points (1 min)/6 points (5 min)). He was immediately intubated and transferred to the neonatal intensive care unit (NICU).

**Figure 3:**
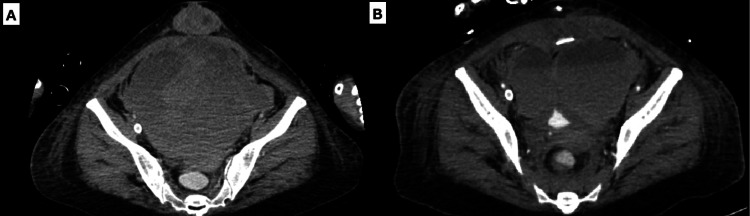
Postoperative hematoma on ECMO with abdominal CT A: On POD 9, a hematoma had formed. The size of the hematoma was approximately 15 cm. We were not able to identify the culprit blood vessels using abdominal contrast-enhanced CT. B: On POD 10, a hematoma formed again. We were able to identify the culprit blood vessels using abdominal contrast-enhanced CT. ECMO, extracorporeal membrane oxygenation; CT, computed tomography; POD, postoperative day

While the P/F ratio of the patient improved temporarily to 309 after the emergency cesarean section, it dropped rapidly to 85, and VV-ECMO was initiated with an unfractionated heparin drip (3,1200U/day) on postoperative day (POD) 1 (Figure 1B). Ultrasonotomography revealed a hematoma starting from the wound area and extending to 8 cm above the uterus, caused by the heparin infusion. With the extension of the hematoma, resulting in urinary tract obstruction and progression of anemia, a laparotomy was performed (Figure 1C) (Figure 3A).

The hematoma, localized between the subcutis and peritoneum, was evacuated. The main hematoma was located under the fascia. Subsequently, the hematoma reformed the next day (Figure 3B), and hemostasis was achieved with interventional radiology. Considering the risk of bleeding, the heparin infusion was stopped after the laparotomy. After stopping the heparin infusion, the circuit required replacement. After 14 days of ECMO therapy, respiratory function improved, allowing successful weaning off VV-ECMO therapy (Figure 1D). The patient’s hemodynamic status was unchanged, and she was extubated after 21 days of mechanical ventilation.

Although her respiratory function remained stable, rehabilitation for walking, swallowing, and vocalization was required. She was transferred to the general ward six days after extubation. The patient was discharged without any oxygen requirements or complications 54 days after the cesarean delivery.

The neonate was cared for in the NICU, extubated 2 days after delivery, and ultimately discharged at 40 weeks and 2 days corrected age from our institution 79 days later without any complications. He had no laboratory or clinical SARS-CoV-2 infection.

## Discussion

We report a case of severe COVID-19 pneumonia in a pregnant woman who was placed on VV-ECMO following an emergency cesarean section. Considering the complications of ECMO, delivery should be performed prior to initiation of ECMO in the third trimester of pregnancy. The P/F ratio may be useful in guiding an appropriate decision regarding delivery after endotracheal intubation.

Before the COVID-19 pandemic, there were various reports of pregnant women treated with ECMO. Zhang et al. have reported maternal and fetal survival rates of 77.2% and 69.1%, respectively, with ECMO use during pregnancy [12]. In this report, it is critical to note that ECMO for pregnant patients requires the balancing of risks and benefits for both the mother and fetus.

To date, there have been two cases of continuation of the pregnancy in the third trimester in women treated with ECMO. In both cases, there were severe complications of ECMO. In one case, concern for hemolysis, elevated liver enzymes, and low platelets (HELLP) syndrome or abruptio placentae after the initiation of ECMO resulted in an emergency cesarean section [10]. In the other case, there was recannulation after weaning off ECMO and delivery [11]. In addition, there have been severe complications such as acute kidney failure and disseminated intravascular coagulation. Consequently, careful consideration is required regarding whether ECMO can be performed safely in the third trimester of pregnancy.

In previous reports, the most common complication of ECMO was hemorrhage [13-18]. Naoum et al. have reported that 18.4% of pregnant women undergoing ECMO developed mild to moderate bleeding, and 13.4% had severe bleeding requiring surgical intervention [17]. Furthermore, an increased risk of fetal cerebral hemorrhage has been reported [18]. These complications can be fatal for the mother and fetus because it is difficult to perform an extremely urgent cesarean section in the setting of COVID-19 infection. Accordingly, the continuation of pregnancy in the third trimester with women requiring ECMO may not be optimal for the mother and fetus.

The Extracorporeal Life Support Organization (ELSO) guideline recommends the initiation of ECMO at a P/F ratio of <80 mmHg for >6 hours and a P/F ratio of <50 mmHg for >3 hours in severe COVID-19 infection [19]. Delivery should occur before the recommended criteria are applied. By considering delivery when the P/F ratio worsens after the initiation of endotracheal intubation, we can decide the appropriate timing of delivery.

Additionally, Cunningham et al. have reported that the initiation of ECMO within seven days after the initiation of endotracheal intubation provides better outcomes [20]. Accordingly, it may be necessary to decide on the initiation of ECMO within at least seven days after initiation of endotracheal intubation for better outcomes for mother and fetus, in case there has been no improvement in the P/F ratio.

After the initiation of endotracheal intubation, it is crucial to prepare for delivery, considering the possibility of the initiation of ECMO. In this case, prednisolone was changed to dexamethasone for fetal lung maturation and MgSO4 was used for neuroprotection.

Considering the risks and benefits of ECMO for the mother and fetus in third-trimester pregnancies, ECMO should be initiated after delivery for better outcomes. In this case, the mother and baby were discharged home without any complications. In third-trimester pregnancies with severe COVID-19 infection, the timing of delivery and initiation of ECMO has been controversial. The P/F ratio may be useful in assisting with appropriate decision-making regarding delivery and the initiation of ECMO.

It is critical to prepare for delivery after endotracheal intubation in third-trimester pregnancies.

## Conclusions

ECMO can be considered a useful therapy for COVID-19 in postpartum. Considering the complications of ECMO, it is particularly important to prepare for delivery immediately after endotracheal intubation in third-trimester pregnancy.
